# Emerging Technologies for Oral Peptide Delivery: From Bioinspired Systems to Smart Device-Assisted Drug Delivery

**DOI:** 10.3390/ph19091328

**Published:** 2026-08-23

**Authors:** Sara Vasović, Lucija Vasović, Nikola Martić, Somyot Chirasatitsin, Velibor Vasović, Saša Vukmirović, Nebojša Pavlović

**Affiliations:** 1 Faculty of Medicine, University of Novi Sad, 21000 Novi Sad, Serbia; 902031d25@mf.uns.ac.rs (S.V.); vasovicl720@gmail.com (L.V.); 2 Institute for Pulmonary Diseases of Vojvodina, 21204 Sremska Kamenica, Serbia; 3 Department of Pharmacology, Toxicology and Clinical Pharmacology, Faculty of Medicine, University of Novi Sad, 21000 Novi Sad, Serbia; nikola.martic@mf.uns.ac.rs (N.M.); velibor.vasovic@mf.uns.ac.rs (V.V.); 4 Department of Biomedical Sciences and Biomedical Engineering, Faculty of Medicine, Prince of Songkla University, Hat Yai 90110, Thailand; somjot.c@psu.ac.th; 5 Department of Pharmacy, Faculty of Medicine, University of Novi Sad, 21000 Novi Sad, Serbia; nebojsa.pavlovic@mf.uns.ac.rs

**Keywords:** peptide drugs, oral delivery, formulation strategies, bioavailability, gastrointestinal barriers

## Abstract

Peptide therapeutics occupy a unique position between small organic compounds and large protein biomolecules, combining high specificity, strong pharmacological efficacy, and favourable safety profiles. Consequently, they have emerged as important therapeutic agents for a wide range of diseases, including metabolic and oncological disorders. However, oral administration of peptide drugs remains a major challenge due to extensive enzymatic degradation, low intestinal permeability, mucus entrapment, and presystemic metabolism within the gastrointestinal tract. This review provides a comprehensive overview of contemporary strategies for improving oral peptide delivery, with special emphasis on emerging pharmaceutical formulation technologies, bioinspired delivery systems and ingestible device-assisted approaches. A qualitative literature search was conducted using major scientific databases and included relevant publications available up to May 2026. The analysis identified the main barriers responsible for low oral bioavailability of peptide drugs, as well as promising approaches to overcoming these obstacles, including peptide modification, enzyme inhibition, permeation enhancement, mucolytic strategies, and advanced carrier systems. Special attention is given to multifunctional carrier systems, ingestible medical devices and bile acid-inspired technologies as emerging directions in oral peptide delivery. The convergence of pharmaceutical sciences, bioinspired formulation strategies and biomedical engineering is expected to accelerate the clinical translation of oral peptide formulations and enable their therapeutic potential to be fully exploited.

## 1. Introduction

### 1.1. Peptides as Active Pharmaceutical Ingredients

**Figure 1:**
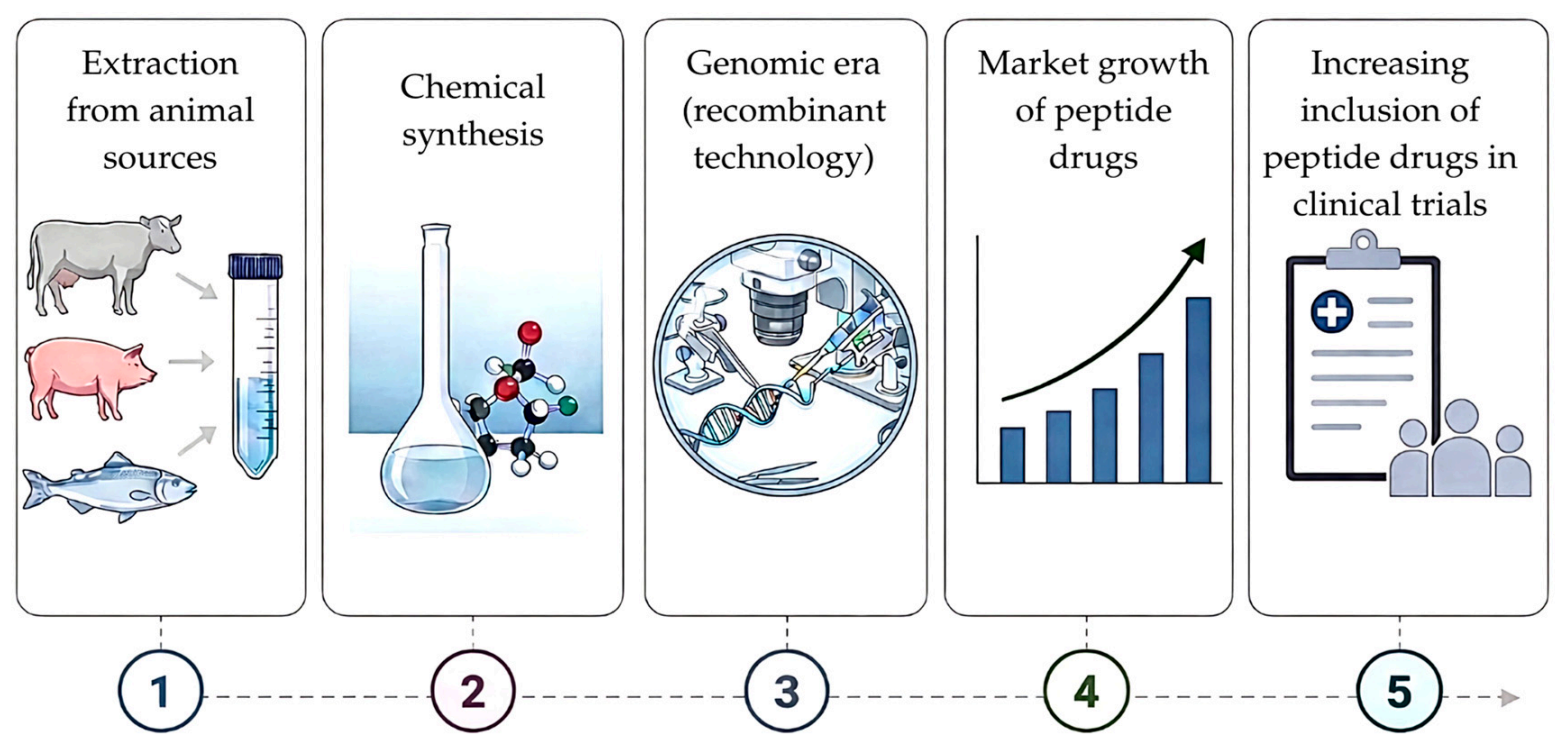
Milestones in peptide drug development (note: the structural layout for this figure was initially drafted by the authors in Canva, with assistance from Gemini and Microsoft Copilot for final illustration generation, and subsequently verified and edited by the authors for scientific content).

Peptides are small-sized biomolecules composed of short chains with up to 50 amino acid residues and occupy a distinctive position between small organic compounds and large protein biomolecules due to their size, therapeutic potential and biochemical specificity [1,2,3,4,5]. Beginning with the clinical use of insulin, peptide therapeutics significantly transformed traditional therapeutic approaches throughout the 20th century, and their considerable effectiveness contributed to the growth of the global peptide therapeutics market [6]. Excellent target specificity and good tolerability allow these therapeutics to achieve desired effects even at low doses [7]. In addition to their targeted activity and regulatory function, peptides also play a significant role in hormone replacement therapy by compensating for insufficient endogenous production [1,8]. The production of peptides for medical purposes has undergone distinct changes over time, reflecting the remarkable progress achieved in this field. Figure 1 highlights the pivotal moments in peptide development. During the first half of the 20th century, peptides such as insulin and ACTH were extracted from animal sources, while during the 1950s the possibility of chemical peptide synthesis based on previously identified amino acid sequences emerged, which led to the production of the first synthetic forms of oxytocin and vasopressin [1,9,10]. In the subsequent period, the isolation of bioactive peptides from exotic sources, such as animal venoms, became a prevailing trend, followed by the genomic era [11]. This era enabled the identification of numerous hormone receptors, causing the focus of interest for both industry and academia to shift towards the discovery of new peptide ligands for these receptors [11]. The transition from the theoretical discovery to the clinical application of synthetic peptides lasted for approximately five decades [12]. Due to the economically unfeasible nature of large-scale peptide production, only peptide hormone analogues that were able to achieve the desired effect at low doses were perceived as commercially viable, which ultimately led to limited interest from the pharmaceutical industry and stagnation in the development of these therapeutics [12]. Conventional approaches that were initially used in peptide synthesis made the process arduous and time-consuming until 1963, when Bruce Merrifield succeeded in accelerating and automating the production process by introducing solid-phase peptide synthesis (SPPS) [10,13,14,15]. The advent of recombinant technology represented an important milestone in peptide drug development, as it enabled the selective expression of genes encoding endogenous human peptides in cell culture systems and successfully reduced the incidence of allergic reactions previously associated with the administration of animal-derived xenogenic peptides [10,12,16]. The approval and widespread clinical adoption of recombinant insulin, together with synthetic gonadotropin-releasing analogues, such as leuprolide and goserelin, demonstrated that peptide-based therapeutics could achieve substantial commercial success [10]. As a result of progress in drug delivery technologies, formulation strategies and peptide synthesis procedures, considerable investments were made in this research domain, which caused a twofold increase in the number of peptides that entered clinical trials between 2000 and 2010 compared to the 1990s [12]. Since peptide therapeutics are used to treat a broad range of diseases, particularly metabolic, cardiovascular and oncological disorders, the past decade has seen a substantial increase in the number of scientific publications in this field [2,17]. So far, the FDA has approved more than 100 peptide-based drugs, while over 1200 candidates are currently undergoing clinical trials worldwide [18]. Considering the increase in investment and research activity in the field of peptide therapeutics, their effectiveness in treating various diseases and the refinement of existing peptide synthesis technologies, the peptide drug market is expected to continue growing in the coming years [12,19,20].

Despite remarkable progress in peptide drug discovery and development, efficient oral administration remains one of the main challenges in peptide therapeutics. Therefore, contemporary pharmaceutical research is focused on overcoming complex gastrointestinal barriers, leading to the development of novel formulation technologies and interdisciplinary drug delivery strategies.

### 1.2. Routes of Peptide Drug Administration

Physicochemical properties of peptide therapeutics, such as their relatively large molecular mass, high hydrophilicity, sensitivity to proteolytic enzymes and fluctuations in pH value, significantly contribute to their low bioavailability [21,22]. In addition to the previously mentioned unfavourable properties of peptides, short residence time and rapid clearance represent additional challenges which dictate that peptides are predominantly administered parenterally (via the intravenous, subcutaneous or intramuscular routes) [2,23,24]. However, the daily parenteral administration required for certain peptide therapeutics, such as insulin, can be problematic because it requires complex administration protocols and frequently causes aversion, concern, fear of needles, discomfort and injection-site reactions, which may eventually lead to suboptimal treatment, poor adherence and disease progression [4,25,26]. The need for parenteral administration makes peptide therapeutics less desirable for chronic treatment requiring long-term medication [1]. Since parenteral application is not favourable among patients, investigation of different routes of peptide administration has begun, while finding an alternative non-invasive route is considered one of the principal goals of biopharmaceutical research [2,21,23,27]. The main difficulties that occur when peptides are administered via alternative routes include the need to overcome all absorption barriers and to achieve satisfactory bioavailability [2]. Therefore, current research is increasingly shifting from conventional formulation approaches toward multifunctional delivery platforms that can simultaneously protect peptides from degradation and promote their intestinal absorption.

Although previous attempts to obtain a suitable oral insulin formulation were not successful, a turning point in this field came with the authorisation of oral GLP-1 agonist semaglutide for the treatment of type 2 diabetes [25,28]. The approval of oral semaglutide represents a major milestone in peptide drug delivery, which has changed the perception that peptides cannot be effectively delivered by the oral route and has stimulated the development of next-generation delivery platforms. In addition to the oral route of administration, many studies are currently focused on developing effective strategies to enhance transdermal, pulmonary, nasal, buccal, vaginal and rectal peptide drug delivery [2,21]. It is believed that the development of these alternative administration routes could pave the way for broader use of peptide therapeutics [8,22].

### 1.3. Registered Peptide Drugs for Oral Use

The introduction of recombinant human insulin, Humulin^®^, into clinical practice in 1981 marked the beginning of the approval and registration of numerous other peptide drugs [29]. Over the years, the role of these therapeutics has expanded, and thus today’s representatives are not solely intended to mimic hormones, as was the case with those that were first registered [13,30]. Development of formulations for oral use plays a crucial role, considering that aversion to parenteral administration appears frequently, especially when it comes to chronic therapy, leading to low adherence [29]. Therefore, some registered peptides and peptide-based drugs are available on the market in formulations for oral use, as shown in Table 1 [2,23,29,31,32,33,34,35,36]. The table includes both systemically absorbed peptide therapeutics and peptide-based drugs with local effects within the gastrointestinal or oropharyngeal tract. Some locally acting antimicrobial products, including vancomycin, colistin and tyrothricin, are structurally more complex glycopeptides, cyclic lipopeptides or mixtures of peptide antibiotics rather than conventional therapeutic peptides.

The successful clinical translation of these peptide formulations for oral use demonstrated that gastrointestinal barriers could be overcome through adequate formulation design. However, future advances are expected to rely on integrating complementary approaches, including peptide engineering, advanced carriers, bioinspired delivery systems and ingestible medical devices, rather than on the application of a single delivery strategy.

## 2. Scope, Novelty and Aim

Several recent review articles have provided comprehensive overviews of the gastrointestinal barriers and formulation approaches relevant to the oral delivery of therapeutic peptides, while others have focused on specific therapeutic areas (e.g., diabetes) or on individual technologies, including nanocarriers, permeation enhancers and ingestible devices. However, the available literature remains largely fragmented between formulation-oriented reviews and technology- or disease-specific analyses. The present review addresses this gap by integrating peptide engineering, biochemical approaches, conventional and advanced carrier systems, bile acid-based technologies and smart ingestible devices within a single framework. In addition to describing their mechanisms, advantages and limitations, the review considers their clinical maturity, manufacturability, safety and regulatory challenges. This integrative approach is supported by the multidisciplinary and international composition of the research team, which brings together expertise in pharmaceutical technology, molecular pharmacology and biomedical engineering.

Accordingly, this review aimed to provide a comprehensive overview of current knowledge and recent advances in oral peptide delivery, with special emphasis on emerging pharmaceutical technologies, bioinspired drug delivery systems and smart ingestible device-assisted approaches designed to overcome gastrointestinal barriers. Furthermore, the review discusses interdisciplinary strategies designed to improve peptide stability, intestinal permeability and systemic bioavailability, together with their translational potential for future clinical application.

## 3. Methods

The retrieved literature was critically evaluated to identify the main barriers to oral peptide delivery, as well as contemporary pharmaceutical strategies and interdisciplinary technologies aimed at improving peptide stability, intestinal permeability and oral bioavailability. The evidence was critically appraised with consideration of study design, level of evidence (preclinical vs. clinical), and reported limitations. Evidence from in vitro and animal studies was interpreted as preclinical and not directly extrapolated to clinical efficacy. This manuscript was designed as a narrative review. A literature search was performed using PubMed and Google Scholar to identify original research articles and review papers published in English up to July 2026. Publications addressing oral peptide therapeutics, gastrointestinal barriers, peptide engineering, formulation technologies, bioinspired drug delivery systems and ingestible medical devices were considered. Original studies were primarily used to summarise experimental evidence and quantitative findings, whereas review articles were used to provide broader background information, identify emerging research trends and support critical discussion of the field. References were managed using Zotero, where duplicate records were removed prior to evaluation. The final selection of publications was based on their scientific relevance to the scope of this narrative review rather than on predefined systematic review criteria.

## 4. Results and Discussion

### 4.1. Barriers for Gastrointestinal Absorption

**Figure 2:**
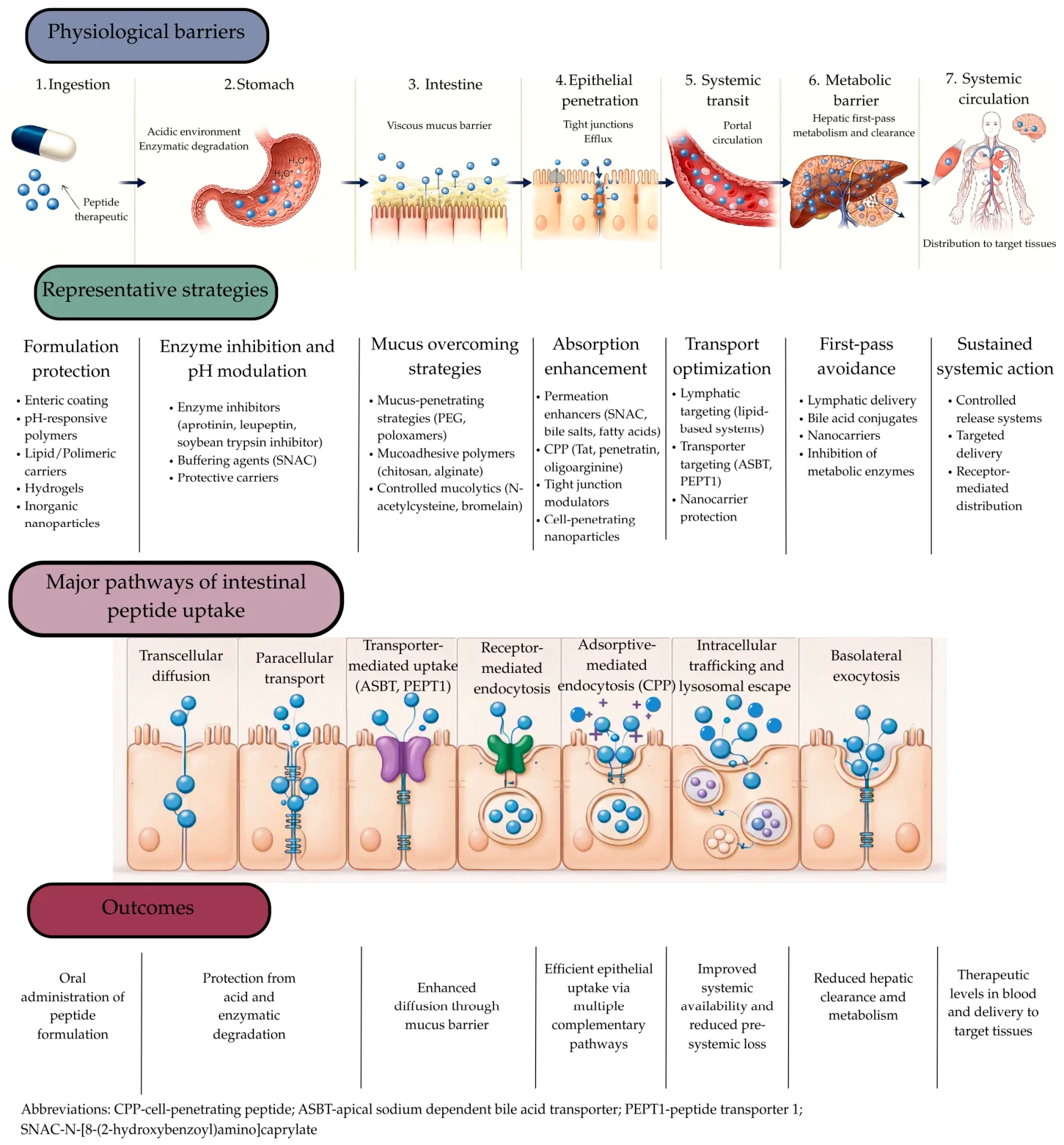
Mechanistic journey of an orally administered peptide through the gastrointestinal tract. The schematic illustrates the sequential fate of an orally administered peptide from ingestion to systemic circulation. Major gastrointestinal barriers, including acidic pH, proteolytic degradation, mucus entrapment, epithelial tight junctions, efflux and hepatic first-pass metabolism, are linked to representative formulation strategies designed to overcome them, such as enteric coatings, enzyme inhibitors, mucus-penetrating systems, permeation enhancers, transporter-targeted uptake, cell-penetrating peptides, bile acid-based systems and lymphatic delivery. The figure also highlights the principal cellular pathways involved in intestinal uptake, including paracellular transport, transcellular absorption, endocytosis and basolateral exocytosis (Note: Individual graphical elements within this figure were generated using ChatGPT (Mobile version 1.2026.223), Gemini (Mobile version 1.0.946899233) and Microsoft Copilot (Mobile version 30.0.440814001) based on author prompts. The elements were subsequently assembled, annotated with text, and verified for scientific accuracy by the authors using Canva (Mobile version 2.373.0)).

**Figure 3:**
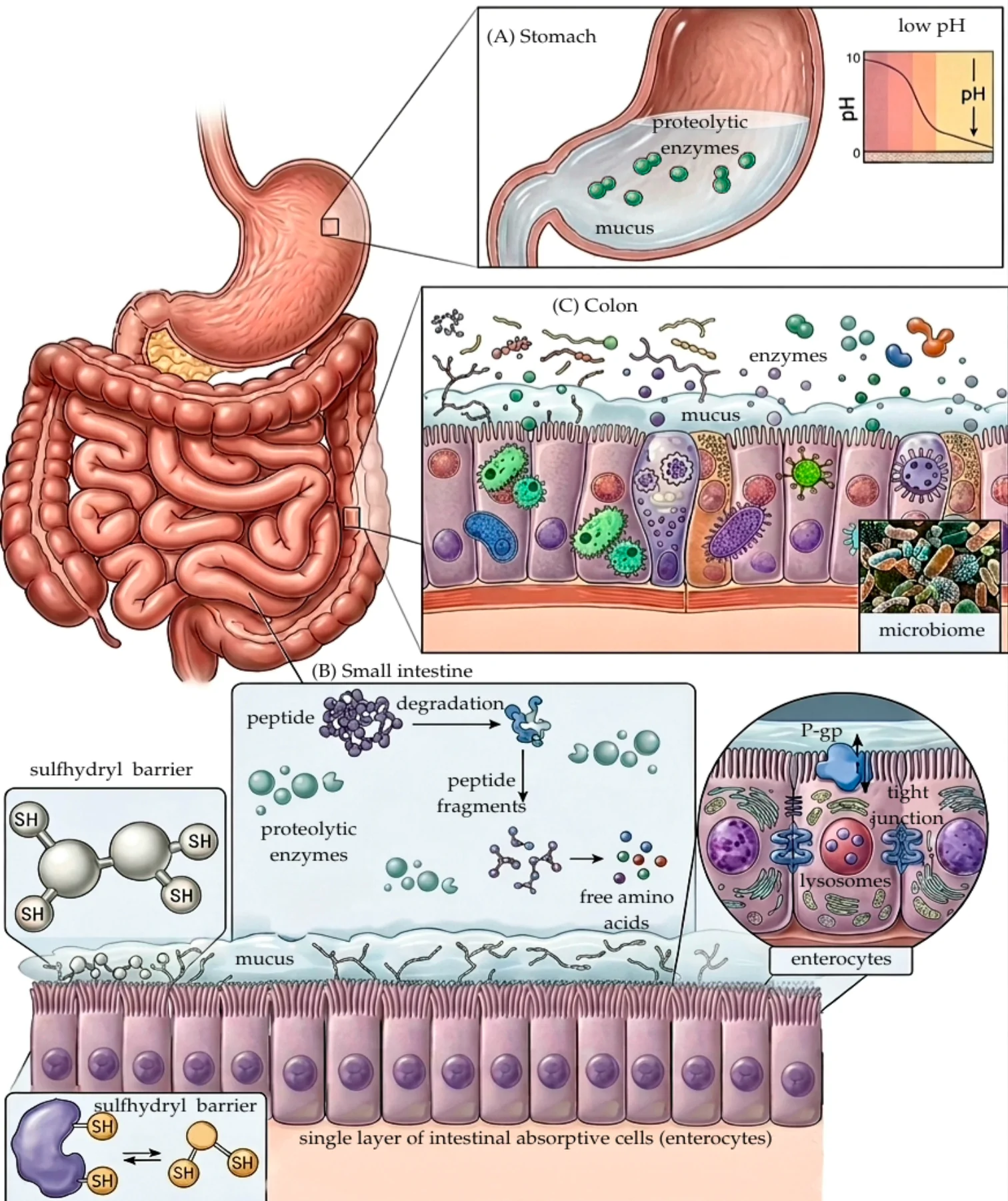
Main gastrointestinal barriers in oral peptide delivery. Schematic representation of regional barriers: (**A**) Stomach: Low pH, mucus and proteolytic enzymes. (**B**) Small intestine: Mucus, proteolytic enzymes, enterocyte monolayer, lysosomes, tight junctions, P-glycoprotein efflux pumps and sulfhydryl barrier. (**C**) Colon: Mucus layer, gut microbiome and microbial enzymes (Note: The structural layout for this figure was initially drafted by the authors in Canva, with assistance from Gemini and Microsoft Copilot for final illustration generation, and subsequently verified and edited by the authors for scientific content).

**Figure 4:**
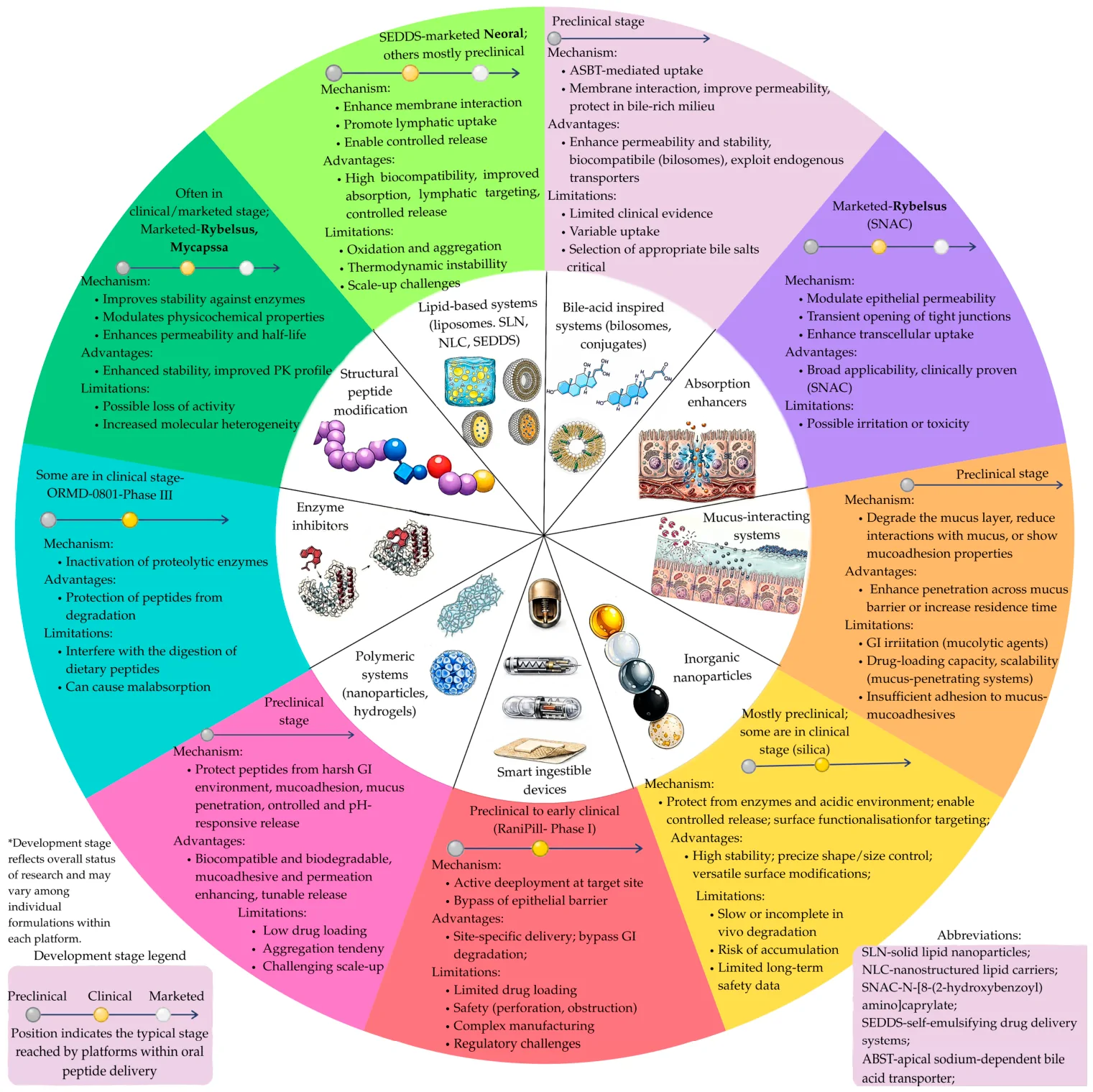
Comparative translational overview of advanced oral peptide delivery technologies. The schematic compares the principal technological approaches investigated for oral peptide delivery, including structural peptide modification, absorption enhancers, enzyme inhibitors, mucus-interacting systems, lipid-based systems, polymeric systems, inorganic nanoparticles, bile acid-inspired systems and smart ingestible devices. For each platform, the figure summarises the main mechanism of action, representative examples, approximate stage of development and principal advantages and limitations. Representative clinically translated products, such as Rybelsus^®^, Mycapssa^®^ and Neoral^®^, are included to highlight the current status of successful oral peptide delivery strategies (Note: Individual graphical elements within this figure were generated using Gemini and Microsoft Copilot based on author prompts. The elements were subsequently assembled, annotated with text, and verified for scientific accuracy by the authors using Canva).

**Figure 5:**
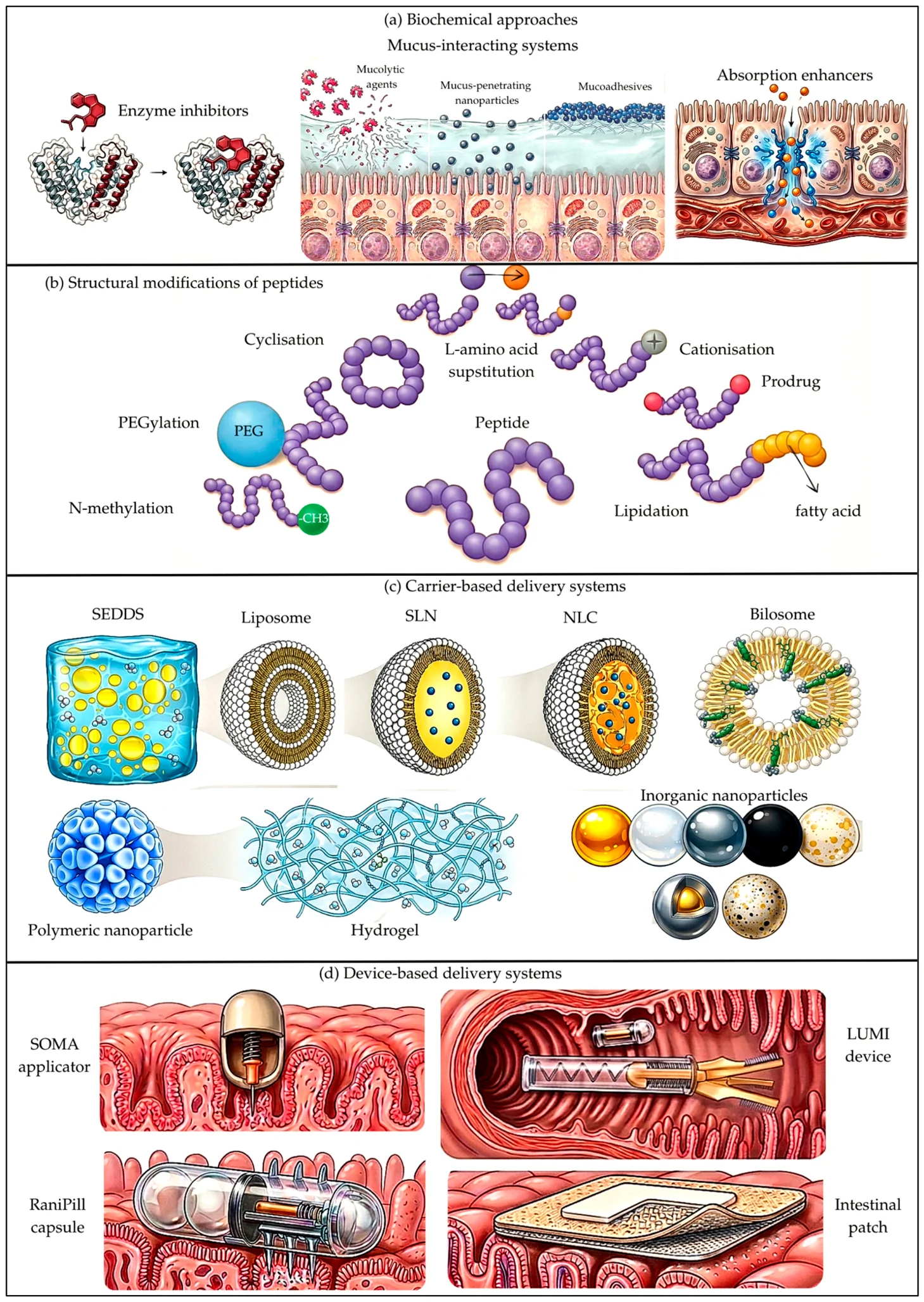
Strategies for improving oral peptide delivery. (**a**) Biochemical approaches including enzyme inhibitors, mucus-interacting systems (mucolytic agents, mucus-penetrating nanoparticles, and mucoadhesives) and permeation enhancers. (**b**) Carrier-based systems such as self-emulsifying drug delivery systems (SEDDSs), liposomes, solid lipid nanoparticles (SLNs), nanostructured lipid carriers (NLCs), bilosomes, polymeric nanoparticles, hydrogels, and inorganic nanoparticles. (**c**) Structural modifications of peptides, including cyclisation, PEGylation, lipidation, cationisation, prodrug formation, N-methylation and amino acid substitution, used to improve peptide stability, permeability, and pharmacokinetic properties. (**d**) Device-based delivery systems, including the SOMA applicator (self-orienting millimetre-scale applicator), RaniPill capsule (robotic capsule), LUMI device (luminal unfolding microneedle injector), and intestinal patches (Note: Individual graphical elements within this figure were generated using Gemini and Microsoft Copilot based on author prompts. The elements were subsequently assembled, annotated with text, and verified for scientific accuracy by the authors using Canva).

The oral route of administration is considered the route of choice for various drugs because it is painless, non-invasive and practical; it simulates the natural route of food intake, reduces the risk of immunogenicity, does not require additional devices for application, enhances adherence and increases cost-effectiveness compared with injections [2,23,28,31]. Considering that the oral route of administration is preferred by patients, extensive studies directed towards ensuring proper oral delivery of peptides such as calcitonin, insulin, human growth hormone and parathyroid hormone have been conducted [2]. Orally administered peptides follow the same gastrointestinal pathway as nutrients, during which they face challenges such as degradation and extensive metabolism prior to absorption into the systemic circulation [2]. The mechanistic journey of orally administered peptides through the gastrointestinal tract, including the major barriers and formulation strategies to overcome them, is illustrated in Figure 2. Gastrointestinal absorption of ingested peptide drugs is affected by interindividual pharmacokinetic variability, differences in food intake and concomitant medication use, the presence of mucus and efflux pumps, exposure to extreme pH values, the sulfhydryl barrier, inadequate solubility, hydrophilicity, high molecular weight, susceptibility to enzymatic degradation and poor intestinal permeability [2,23,28,46]. The major components constituting the gastrointestinal barrier are summarised in Figure 3. The existence of a pH gradient along the gastrointestinal tract has a substantial impact on the state of ionisation, chemical stability, biological activity and absorption of peptide drugs, as well as the extent to which the degradation enzymes will be active [23]. The acidic environment in the stomach induces denaturation, alters spatial conformation and contributes to inactivation in the presence of proteolytic enzymes, whereas the gradual increase in pH along different segments of the intestine reduces peptides’ susceptibility to degradation [23,47,48]. This situation is highly complex because pH value may vary under the influence of nutrition and the existence of certain pathological conditions [49]. Gastric emptying and intestinal motility have an impact on peptide gastrointestinal retention, residence time in different gastrointestinal segments, as well as degree of exposure to an unfavourable microenvironment, thereby affecting peptide degradation and absorption [6]. In the proximal parts of the gastrointestinal tract, ingested peptides encounter salivary digestive enzymes, such as amylase and lipase, which are indifferent towards peptides, only to be subsequently exposed to a lower pH value and to digestion by proteolytic enzymes, pepsin and cathepsin [7,21,50]. Further degradation takes place in the intestines via endopeptidases (trypsin, chymotrypsin, elastase) and exopeptidases (carboxypeptidase A and B) produced as a result of the exocrine function of the pancreas, as well as via enzymes located on the enterocyte brush border that are mainly responsible for degradation of short peptides to free amino acids [3,51,52]. Luminal degradation by pancreatic enzymes contributes to the overall peptide breakdown, ranging from 5% to a maximum of 20%, while brush border and intracellular enzymes of the enterocytes are responsible for the major part of peptide degradation, with the additional influence of gut microbiome enzymes [6,23,27]. The gut microbiome can initiate fermentation, deamination, and decarboxylation, along with proteolysis, and the products of their metabolic activity can change pH value and microenvironmental enzymatic activity, affecting the stability and absorption of peptide drugs [6]. Endocytosis leads to the formation of endosomes through which peptides are taken up into the cell, and then as endosomes mature and fuse with lysosomes, peptides become exposed to a more acidic medium, which is suitable for activation of various hydrolytic enzymes, including cathepsins [6,53,54]. Although some peptide therapeutics manage to avoid the endosomal compartment, they still become the target of the high catalytic activity of cytosolic proteases such as proteasome and aminopeptidases [6]. For orally administered drugs to reach the systemic circulation, it is necessary that they overcome the barrier formed by a single-layer epithelium composed of enterocytes that are separated by tight junctions made of transmembrane proteins like occludins, claudins, tricellulin and junctional adhesion molecules [25,35,55]. They form very dynamic structures directly connected to the cytoskeleton, and their role is to regulate intestinal permeation of molecules [23,25]. The high hydrophilicity of peptides disrupts their transcellular transport while their size hinders paracellular passage through narrow intracellular spaces, leading to insufficient absorption [56]. The mucus barrier, produced by goblet cells, is a coherent three-dimensional gel-like matrix composed of two layers, one of which consists of mucin, glycolipids, and glycoproteins and adheres directly to the epithelial surface, while the other, which has a less dense consistency, forms the surface layer that undergoes constant renewal through continuous secretion and removal [32,34,36,51,57]. Mucus represents both a diffusion and a protective barrier whose main role is to protect epithelial cells from mechanical damage, enzymatic degradation, penetration and invasion of pathogens [6,34]. It can interfere with absorption of peptide therapeutics due to its coherent viscoelastic structure that acts as a physical barrier, but also through electrostatic interactions of negatively charged mucin fibres with electropositive peptides [6,23]. These interactions are possible due to the presence of negatively charged residues of sialic acid and sulphate groups located on the oligosaccharide side chains of mucin that attract and bind positively charged peptides, while further reduction in peptide diffusion is dependent on hydrophobic interactions with mucus [6,7,52]. Components of the sulfhydryl barrier, such as thiol groups and disulfide bonds coming from endogenous glutathione, mucus glycoproteins, dietary proteins and food rich in antioxidants with cysteine domains, are capable of interacting with the thiol groups and disulfide bonds of the peptide drugs, leading to their intermolecular binding [52]. Even if these therapeutics manage to enter enterocytes, they can still be transported back into the intestinal lumen through the activity of ATP-binding cassette (ABC) transporters such as P-glycoprotein (P-gp), which is highly expressed in the ileum and colon [6,49,53]. P-gp-mediated efflux can lead to a reduction in intracellular peptide concentration and thereby contribute to their low bioavailability [35,49,58,59]. Those peptides that succeed in overcoming the epithelial barrier enter the portal circulation, through which they are delivered to the liver, where metabolic deactivation takes place [3,58,59]. All the previously mentioned physical and biochemical barriers of the gastrointestinal tract have stimulated intensive research directed towards overcoming low oral bioavailability, which resulted in the discovery and development of the different formulation strategies and innovative technological approaches displayed in Table 2 and Figure 4 and Figure 5. These strategies are predominantly based on two main approaches, one of which involves peptide protection from harsh gastrointestinal conditions while the other focuses on enhancing permeability through the intestinal membrane [23]. Collectively, these barriers demonstrate why a single formulation strategy is rarely sufficient to achieve efficient oral peptide delivery, leading to the development of multifunctional platforms capable of simultaneously addressing multiple gastrointestinal barriers.

### 4.2. Contemporary Strategies for Improving Oral Peptide Delivery

#### 4.2.1. Structural Modifications of Peptides

Structural peptide modifications are intended to enhance the resilience of peptides to enzymatic degradation and improve their membrane permeability through cyclisation, PEGylation, lipidisation, substitution of natural L-amino acids, N-methylation, cationisation and prodrug design. Cyclisation of peptides increases their stability and decreases vulnerability to degradation by removing exposed N- and C- terminals, which are particularly susceptible to enzymatic decomposition, through their association via disulfide, lanthionine, hydrazine, dicarboxylic and lactam bridges [25,59,96]. This type of modification results in the formation of more rigid structures which possess greater proteolytic resilience, reduced flexibility and limited capability to form intermolecular hydrogen bonds [34,35,59,60]. Simultaneously, cyclic peptides have a greater ability to permeate biological membranes, and they contribute to the improvement in oral bioavailability, which is best illustrated by the examples of successfully developed oral formulations of cyclic peptides such as cyclosporine A and desmopressin [21,25,34]. However, if the modification interferes with the biologically active conformation of the peptide, it may lead to a loss of biological activity [23]. PEGylation is a frequently used form of structural modification which includes covalent conjugation of peptides with polyethylene glycol (PEG), a hydrophilic polymer which is considered to be biocompatible, safe and non-immunogenic [6,21,33,60,65]. This modification increases hydrodynamic volume and molecular weight, thereby reducing renal clearance, and it also influences interaction with plasma proteins [6,61]. Additionally, PEGylation provides a steric protection that hinders the approach of proteolytic enzymes, which leads to a reduction in plasma clearance, increased peptide stability and prolonged retention in circulation [23,53]. Covalent attachment of PEG results in shielding of antigenic epitopes that can contribute to a reduction in immunogenicity and antigenicity [53,60]. Nonetheless, extensive PEGylation may impair binding of peptide therapeutics to relevant receptors, and reduce their cell affinity as well as limit their biological activity [6,7]. In preclinical studies, it has been demonstrated that PEG conjugation may enhance diffusion through the mucus barrier by reducing interactions between peptides and negatively charged mucus components [6,25,52]. Chemical modification of the initial peptide molecule leads to the formation of a prodrug, which usually shows greater stability, solubility, lipophilicity and intestinal permeability [7,63]. Upon its in vivo chemical or enzymatic transformation, an active form of a peptide therapeutic is generated [7,63]. Within the prodrug system, it is crucial to recognise the potential toxicity of the attached moiety, which is released upon activation, while also avoiding premature activation and achieving efficient and predictable activation [6]. Lipidation is a form of post-translational modification that can be achieved through covalent or non-covalent strategies and is intended to produce more stable peptide therapeutics with reduced hydrophilicity and improved oral bioavailability [23,24]. During this process, peptides are conjugated with fatty acids that enhance their penetration through biological barriers and contribute to extending their plasma half-life through stronger binding to albumin and plasma lipoproteins [24,60]. It has been observed that adding medium-chain fatty acids leads to both the promotion of paracellular transport of the peptides that belong to the third group of the Biopharmaceutical Classification System (BCS) and the bypassing of the first-pass effect by stimulating uptake via the lymphatic system [6,21]. Incorporation of lipid modifications into the peptide molecules results in their greater affinity for chylomicrons, which favours their uptake into the lymphatic system while exposure to presystemic metabolism is simultaneously avoided [63]. However, lipidation comes with certain drawbacks, which include decreased water solubility of the modified peptides as well as their reduced affinity for relevant receptors [7]. N-methylation of the peptide bond demonstrates a protective effect against enzymatic degradation, while inclusion of the N-methyl amino acids increases resilience against proteolysis by preventing the formation of hydrogen bonds that are necessary for enzymatic recognition [6,65]. Evidence suggests that formulating drugs in cationic form can increase intestinal penetration due to electrostatic interaction with negatively charged cell membrane glycoproteins and glycosphingolipids [7]. Yet cationisation of peptides can result in increased immunogenicity, rapid clearance and loss of drug activity, and the use of this modification is limited by nonspecific cellular uptake and potential toxicity [7]. Another type of structural modification is the substitution of natural L-amino acids with unnatural ones, such as D-amino acids, which is considered to be promising since it reduces susceptibility to enzymatic degradation [65,66,68]. Greater protease resistance arises as a result of the stereospecificity of the digestive enzymes, which predominantly recognise and hydrolyse L-forms of amino acids [6,34].

#### 4.2.2. Application of Enzyme Inhibitors

Enzymatic degradation is considered a major obstacle to the oral delivery of intact peptides and can potentially be addressed through the simultaneous administration of peptides and enzyme inhibitors [2,68]. Various inhibitors of digestive enzymes, such as aprotinin, FK-448, soybean trypsin inhibitor, and leupeptin, as well as ovomucoids, a new class of enzyme inhibitors isolated from duck and chicken eggs, can be found in formulations for oral peptide delivery [21,26,32,34]. They cause reversible or irreversible enzyme inhibition by binding directly to the enzyme’s active site [23,32]. However, extensive use of these inhibitors might result in safety concerns. By causing a deficit of proteolytic enzymes, enzyme inhibitors can interfere with the regular digestion of dietary proteins, leading to their malabsorption [34,69]. Given that the release of digestive enzymes is based on a feedback mechanism, another severe consequence that might occur due to their improper use is pancreatic hypertrophy and hyperplasia [26,33].

#### 4.2.3. Application of Absorption Enhancers

Absorption enhancers are a highly diverse group of chemical agents with the ability to induce transient loosening of tight junctions, to cause a change in mucus viscosity or to modulate fluidity and structural integrity of cell membranes, thereby promoting transcellular or paracellular drug delivery [25,26]. For these purposes, surfactants, fatty acids, bile salt derivatives, chelating agents, chitosan, cholesterol, glycerides, salicylates, nitrogenous-containing heterocyclic compounds, cell-penetrating peptides (CPPs) and aromatic alcohols can be used [23,49,72]. Ideal absorption enhancers should provide effective and transient modification of gastrointestinal epithelial permeability while maintaining an appropriate safety profile and minimal local and systemic toxicity [25]. While transient impairment of tight junction integrity improves intestinal absorption of peptide therapeutics, prolonged and excessive use of these agents may lead to gastrointestinal inflammation and systemic endotoxemia due to increased absorption of toxins and pathogens caused by the compromise of intestinal mucosal integrity [26,34,69,73]. During preclinical trials, numerous permeation enhancers were examined, but only some of them showed a favourable balance between safety and efficacy [25]. Certain absorption enhancers, like EDTA, sodium cholate and sodium dodecyl sulphate, can potentially disrupt epithelial barrier function, causing systemic toxicity [7]. By combining absorption enhancers with different mechanisms of action, it is possible to create a synergistic effect with minimal individual doses while minimising toxicity [25]. Investigation of plant-derived absorption enhancers has indicated their potential to improve intestinal absorption with reduced adverse effects [6]. Pelargonidin, a flavonoid isolated from strawberries, has proven to be a promising candidate for oral peptide delivery [51]. Unlike synthetic absorption enhancers, which disrupt the integrity of the intestinal epithelium and lead to irritation, this compound has shown that with prolonged use it does not cause adverse effects [6]. Absorption enhancers, such as sodium SNAC (N-[8-(2-hydroxybenzoyl)amino]caprylate) and 5-CNAC (8-(N-2-hydroxy-5-chloro- benzoyl)-amino-caprylic acid), turned out to be very successful in promoting an increase in bioavailability, with both demonstrating the potential to enhance transcellular transport without compromising intestinal integrity [7]. A commercially available oral formulation of semaglutide (Rybelsus^®^) contains SNAC as a permeation enhancer [106]. In this formulation, SNAC not only promotes the transcellular absorption of semaglutide across the gastric mucosa via a transient and reversible mechanism, but also protects this peptide with its buffering activity [84,107]. This localised pH elevation inhibits the activation of pepsinogen and prevents semaglutide degradation [4,107]. Furthermore, SNAC is able to diminish intermolecular hydrophobic interactions responsible for peptide oligomerisation, thereby maintaining semaglutide in fast-absorbing monomeric form [4,84]. Cell-penetrating peptides (CPPs) can be derived from viral proteins with the ability to translocate efficiently across cell membranes, or, alternatively, they can be non-viral proteins and small molecules that mainly interact with glycosaminoglycans, promoting uptake of peptide therapeutics via endocytosis [7,25]. These short-chain peptides usually contain up to 30 amino acids, and they are recognisable by the presence of the positively charged residues of arginine and lysine [56,108]. The cationic nature of the CPPs determines their ability to cross the cell membrane by affecting interactions with negatively charged glycosaminoglycans and sialic acid located on the cell surface [56,108,109]. The presence of hydrophobic amino acids, such as tryptophan, within the CPP sequence affects interactions with the membrane’s lipid components, which consequently leads to the promotion of endocytosis [21,108]. The CPP group includes the Tat peptide derived from the HIV-1 Tat (Trans-Activator of Transcription) protein, penetratin and oligoarginine [56,110,111]. Facilitation of peptide drug delivery is achieved either by simple mixing with CPPs or through conjugation with them [21,56,63]. Nanoparticles modified by surface binding of CPPs are sometimes used in order to enhance the absorption of peptide therapeutics through greater intracellular uptake [6]. However, strategies involving CPPs are associated with certain disadvantages, such as the risk of membrane destabilisation, low selectivity and immunogenicity, which limit their potential for clinical application [6,108]. Gastrointestinal instability, toxicity and endosomal entrapment of CPPs can be resolved through application in the form of an enterosolvent capsule, which protects against acidic and enzymatic degradation, enables extended release and reduces the toxic effects of CPPs on the intestinal mucosa [7].

#### 4.2.4. Mucus-Interacting Systems

Mucolytics are compounds that can disrupt the coherent structure of the mucus barrier by degrading intermolecular disulfide bonds, thereby contributing to the increased permeability and greater oral bioavailability of peptide therapeutics [36,63,112]. In experimental models, dithiothreitol, N-acetylcysteine, bromelain and papain are commonly used for this purpose [23]. Nevertheless, a problem may arise if the protective mucus layer is extensively removed, as it can cause harmful exposure of the intestinal epithelium to the damaging effects of enzymes and digestive fluids [23,36,63]. Although both belong to the group of mucus-interacting systems, mucus-penetrating agents offer a less invasive approach to crossing the mucus barrier compared to the previously mentioned mucolytic agents. Mucus-penetrating systems are designed to enable diffusion through the protective layer of mucus and thereby to establish direct contact with the underlying epithelial cells without compromising the integrity of the mucus barrier [75]. By covering the surface of the drug-loaded nanoparticles with nonionic polymers such as low-molecular-weight PEG and poloxamers (mainly the Pluronic class) or with a compact, uniformly distributed layer composed of oppositely charged materials (e.g., chitosan and alginate) providing electrostatic neutrality, it is possible to reduce electrostatic interactions with the mucus components and thereby to enhance the passive penetration of a therapeutic agent across this protective layer [75,76]. Mucus-interacting systems also include those with mucoadhesive properties, which are described in this review as part of polymer-based drug delivery systems and mucoadhesive intestinal patches.

#### 4.2.5. Drug Delivery Systems

Nowadays, drug carrier systems are considered to be as important as the drug itself because they enable controlled drug release, prolonged drug delivery and maintenance of the therapeutic drug concentration in the bloodstream, thereby reducing the frequency of administration, side effects and toxicity [113]. Moving beyond earlier microparticle drug carrier systems, modern drug delivery strategies focus predominantly on nanoparticle therapeutic systems, which have demonstrated an improvement in the bioavailability of orally administered drugs due to their small size, large surface area, effective protection from enzymatic degradation and controlled release [49,53,60,113]. Nanoparticle therapeutic systems can be classified as lipid, polymeric or inorganic nanoparticles, each with specific advantages and limitations.

##### Lipid Therapeutic Systems

Lipid nanoparticles represent a special group of drug delivery systems that includes biocompatible and biodegradable carriers such as liposomes, solid lipid nanoparticles (SLNs) and nanostructured lipid carriers (NLCs) [6,32,80,81,82,83,92,114]. Their lipid composition favours absorption via the lymphatic system and bypassing of the hepatic first-pass effect [36,80,81]. Liposomes are spherical amphiphilic vesicles composed of one or more phospholipid layers that surround an aqueous core suitable for encapsulation of hydrophilic drugs, including peptides [36,92,114]. The attachment of PEG molecules to the surface of liposomes has a protective effect against opsonisation, phagocytosis and degradation in an acidic environment, while simultaneously providing effective cellular internalisation and intestinal permeation compared to conventional forms [34]. The SLN matrix is formed by stabilising solid lipid components upon the addition of surface-active agents, thereby protecting incorporated peptides and controlling their release [59,80,83]. Combining solid and liquid lipids results in the formation of an NLC, a lipid therapeutic system that shows greater capacity for drug encapsulation and a lower crystallisation tendency [6,81]. In addition to the benefits that arise from the application of lipid drug delivery systems, the challenges of their implementation should also be considered, and they include oxidation susceptibility, difficulties in industrial production and aggregation tendency caused by their thermodynamical instability [6,7]. Fluctuations in pH value, along with the presence of enzymes and salts, can also compromise the stability of these drug delivery systems [84]. Alongside the classical lipid nanoparticle formulations, over the past years, self-emulsifying drug delivery systems (SEDDSs), which include self-microemulsifying drug delivery systems (SMEDDSs) and self-nanoemulsifying drug delivery systems (SNEDDSs), have attracted considerable interest due to their ability to improve oral bioavailability [21,49,70]. Upon contact with gastrointestinal fluids, these systems undergo spontaneous emulsification, followed by the formation of an O/W (oil-in-water) emulsion [33,52,92]. SEDDSs are considered suitable due to their simple and cost-effective manufacturing process, while simultaneously showing the ability to enhance penetration of peptide drugs through the mucus barrier and epithelium as well as to protect peptides from presystemic metabolism [6,21,70]. The only approved peptide drug for oral administration formulated using SEDDS technology is cyclosporine A (Neoral^®^), an immunosuppressive drug available as a concentrate that forms a homogeneous single-phase microemulsion upon contact with gastrointestinal fluids and consequently promotes absorption [31,70]. From a translational perspective, lipid-based systems do not represent a uniform category. Lipid nanoparticles (liposomes, SLNs, NLCs) demonstrate well-established biocompatibility and biodegradability, with preclinical studies documenting peptide protection and showing potential for oral peptide delivery. Oxidation, aggregation, thermodynamic instability, and challenges in scaling up the manufacturing process to an industrial level represent significant constraints that complicate the development of these therapeutic systems. Nevertheless, the persistent difficulty of translating preclinical results into reproducible clinical outcomes remains the decisive barrier to their broader clinical application. In contrast, self-emulsifying drug delivery systems (SEDDSs) are characterised by simple and cost-effective manufacturing and have already reached clinical application, with the approved Neoral^®^ formulation serving as a representative example. Overall, these findings highlight that while classical lipid nanoparticles still face significant translational limitations, SEDDSs currently represent the most advanced lipid-based approach with realistic potential for broader clinical application in the future.

##### Polymeric Drug Delivery Systems

Along with lipid systems, polymeric nanoparticles composed of biocompatible and biodegradable polymers such as chitosan, PLGA (polylactic-co-glycolic acid), PLA (polylactic acid), and PHA (polyhydroxyalkanoates) represent a widely investigated platform for oral delivery of peptide therapeutics [6,63,69,109,115]. By incorporating peptides into the polymeric matrix of nanoparticles, protection from unfavourable gastrointestinal conditions is achieved, and through surface modifications of these particles, it is possible to enhance their mucoadhesive properties, facilitate transport across the mucus, reduce clearance, improve intestinal uptake and increase selectivity towards relevant receptors and transporters [6,69,116]. Nanomaterials based on polysaccharides offer significant advantages due to their biocompatibility, biodegradability, safety, pH-responsive drug release, and ability to interact with mucus, thereby leading to an extended residence time and consequent drug absorption [6,21,23,87]. Owing to their wide availability, simple preparation, safety, biodegradability and excellent mucoadhesive properties, marine-derived polysaccharides, chitosan and alginate have emerged as preferred matrices within modern drug delivery platforms [84,87,114,117,118,119,120]. By interacting with sialic acid residues of the mucus surface, chitosan promotes mucoadhesion and, at the same time, demonstrates the ability to enhance peptide penetration through tight-junction loosening [21,53,59]. Recently, modified forms of chitosan, such as N-trimethyl chitosan (TMC), as well as derivatives formed through covalent attachment of thiol groups, have gained increasing attention due to their more pronounced interaction with mucus [120]. The application of systems with mucoadhesive properties encounters certain limitations, including reduced adhesion time caused by constant mucus renewal, the difficulty of aligning mucoadhesion with drug release kinetics, as well as interindividual differences in mucus composition [6]. Advanced approaches to peptide delivery include pH-responsive polymer carriers such as hydrogels, which contribute to greater peptide stability in the gastric environment and enable controlled release of therapeutics in the intestine [121]. Hydrogel formulations primarily contain an aqueous phase and a cross-linked polymer, with a peptide drug embedded into this matrix [25,114]. These systems can alter their structural, mechanical and swelling properties in response to changes in microenvironmental factors, and their high water-binding capacity permits efficient incorporation of hydrophilic drugs, including peptides [7,109]. The major disadvantages associated with the application of hydrogels include rapid degradation upon exposure to larger amounts of intestinal fluid and physical and chemical instability [7]. There has been a growing interest in pH-responsive hydrogels composed of natural biopolymers like alginates and chitosan, since many synthetic polymers have proven to be immunogenic, and the process of peptide encapsulation into their matrix demands extreme conditions that could cause peptide denaturation [88,89,90,91]. By covalently modifying chitosan and alginate, it is possible to tailor their physicochemical properties to meet the specific requirements for oral administration [122,123]. For the development of orally administered peptide formulations, pH-responsive chitosan–alginate hydrogel complexes are also being studied [118]. The concept of the polyelectrolyte complex has gained acceptance since it successfully overcomes the limitations of pure chitosan and alginate forms [117,118]. Attention has also been directed towards nanoparticle drug carriers prepared by the ionic gelation technique using alginate and chitosan as polymers (CANPs—chitosan/alginate nanoparticles) [87]. CANPs feature strong electrostatic attraction between amino groups of chitosan and carboxyl groups of alginate, resulting in the formation of a gel-like structure at low pH values, contraction of the cross-linked polymer network and protection of encapsulated peptides from harsh gastric conditions [87,124]. Despite numerous advantages, polymeric drug delivery systems are accompanied by several limitations, including a tendency for aggregation, small drug encapsulation capacity and challenging scale-up process [6].

Collectively, both polymeric nanoparticles and hydrogel systems remain at the preclinical stage with comparable translational maturity. Their specific limitations differ, with polymeric nanoparticles constrained by aggregation, low drug-loading capacity, scalability issues, and the persistent difficulty of translating preclinical findings into clinical outcomes, while hydrogels are affected by rapid degradation and physicochemical instability. Neither platform has yet demonstrated clear superiority. The use of natural polysaccharides, such as chitosan and alginate, represents a future direction for the development of these delivery systems, as it provides biocompatibility, biodegradability, and a favourable safety profile. Accordingly, strategic refinement of both nanoparticle and hydrogel formulations, together with optimisation of manufacturing methods, will be crucial to overcoming existing limitations and to supporting progress toward clinical translation.

##### Inorganic Particles

Inorganic particles are one category of peptide delivery systems that have proven stable in acidic environments as well as in enzyme-rich media [21]. For the oral delivery of peptide drugs, nanocarriers based on silica, selenium, gold, titanium dioxide, zirconium phosphate, and calcium phosphate are being considered [21,32,92,93]. Calcium phosphate nanoparticles stand out for their biocompatibility, biodegradability and physicochemical adaptability, which make them promising platforms for oral peptide delivery [93]. Certain advantages are associated with the use of silica, gold and carbon nanoparticles regarding the feasibility of precise control over their size and shape as well as the potential for implementing surface modification [6]. Mesoporous silica nanoparticles are regarded as good candidates for drug delivery with a great capacity for peptide encapsulation along with controlled release and protection against enzymes, while their modified surface improves peptide bioavailability and plays a role in directing them towards the target tissue [84,94]. Some scientific publications suggest the promising potential of gold nanoparticles in peptide drug delivery, especially those with ultra-small dimensions up to 2 nm, which have relatively prolonged circulation times and enhanced ability for tissue penetration [7]. Carbon nanocarriers are being increasingly investigated, owing to their ability to deliver drugs like insulin to target cells and their unique potential to induce penetration across the cell membrane, which makes them a suitable system for intracellular delivery of therapeutics [125]. Even though these systems show stability under physiological conditions, they encounter limitations in terms of clinical application as a result of their slow and incomplete in vivo degradation and risk of tissue accumulation, which necessitate additional investigations aimed at evaluating their safety [32,63].

Although inorganic nanoparticles are resistant to gastrointestinal conditions and capable of transporting peptides, most have not progressed beyond preclinical investigation. Silica-based nanoparticles for oral insulin delivery, developed by Oshadi Drug Administration, stand out as an exception. They have reached Phase II clinical trials and are currently the most clinically advanced inorganic nanoparticle system for oral peptide delivery. Despite demonstrated clinical progress, silica-based nanoparticles still require rigorous evaluation to confirm both their therapeutic effectiveness and long-term safety. Meanwhile, preclinical evaluation of other inorganic nanoparticles requires the development of reliable experimental and predictive models to better assess their properties and safety. For the majority of inorganic systems, slow degradation, bioaccumulation, and toxicity remain unresolved problems that hinder further development. Accordingly, the further advancement of inorganic nanoparticles for oral peptide delivery will require the development of reliable models capable of providing a comprehensive evaluation of their efficacy and safety profiles.

##### Smart Ingestible Medical Devices

The concept of drug delivery via microneedles has been studied over the past two decades for various medical applications, ranging from transdermal drug delivery to vaccine delivery [70,98]. This technology was also adapted for the delivery of peptide drugs with low oral bioavailability, and it is crucial to understand the impact of this approach on gastrointestinal mucosa, since it can be invasive and it may increase the risk of mucosal perforation [70,97]. The literature emphasises the importance of developing microneedles that can dissolve upon contact with the gastrointestinal mucosa and thus reduce the risk of perforation [23]. Furthermore, when applying these systems, potentially significant fluctuations and unpredictability of pharmacokinetic parameters that arise from interindividual variations in peristalsis must be considered [70]. Among the biodegradable ingestible microneedle-based devices intended for oral peptide delivery, LUMI (luminal unfolding microneedle injector) and SOMA (self-orienting millimetre-scale applicator) stand out. The LUMI device is an osmotically controlled system in the form of a two-compartment capsule coated with a pH-responsive polymer that dissolves at pH ≥ 5.5 and prevents premature activation [96,97]. When the capsule reaches the small intestine and the coating dissolves, the osmotic gradient draws intestinal fluid inward and causes the softening of the supporting polymeric element that keeps the spring compressed until activation [70,126]. Consequently, the spring is activated, mechanically expelling a biodegradable three-armed element whose expansion pushes the microneedle patches directly against the intestinal wall and enables drug penetration [25,36,70]. Owing to its specific geometry and self-orienting ability, the SOMA applicator orients itself so that it settles into a stable position on the gastric wall and thereby effectively opposes the dynamic impact of external factors such as fluid flow and peristaltic movements [7,32,96,98]. This ingestible device uses a spring-based mechanism which activates upon entry of gastric fluid, leading to the release and insertion of a drug-filled millipost into the gastric mucosa [32,127]. Studies conducted so far have shown that it is possible to achieve successful delivery of peptide therapeutics like insulin without compromising gastric structural integrity [25,36]. The limitations of these two devices are related to limited drug-loading capacity, whereby increasing the dimensions of the microneedles can improve the loading capacity, along with the increased risk of gastrointestinal perforation [7,23]. To examine the degree to which LUMI and SOMA can be safely and reproducibly directed towards certain gastrointestinal regions, it is necessary to conduct additional investigation [25]. For oral peptide delivery, Rani Therapeutics has developed RaniPill^®^, a hydroxypropylmethylcellulose capsule coated with an enteric polymer, which selectively dissolves in the alkaline medium of the jejunum [25,70,84]. The dissolution of the coating layer initiates carbon dioxide generation, leading to the expansion of the microneedle device’s inner balloon, which results in systemic drug delivery due to the positioning of the soluble sucrose microneedles against the intestinal epithelium and their injection into the intestinal wall [25,32,33,36,70]. However, to gain better insight into the performance of this device, further investigation is required [70]. Another microneedle-based concept for oral peptide delivery involves magnetically controlled microneedles. These are ingestible devices that contain an enteric-coated capsule, drug-filled microneedles, along with an integrated magnetic substrate which, under the influence of an externally applied magnetic field, directs penetration of microneedles through the intestinal wall [23,58,128]. As alternative approaches for the oral delivery of peptide therapeutics, intestinal patches and microreservoirs (microcontainers) are considered [23]. Intestinal patches possess a small reservoir which physically protects the incorporated drug from local enzymatic degradation, and also, due to the presence of a mucoadhesive layer, they enable strong mucosal adhesion and drug positioning near the absorptive epithelium [25,99]. The foundation of the patch is a polymeric matrix impregnated with a drug along with a stabiliser, which functions as a drug reservoir intended for enhancement of intestinal absorption through maintenance of a high concentration gradient in the immediate microenvironment [7,99]. Constraints related to the use of intestinal patches include limited drug loading capacity and limited stability throughout the storage period [7]. Mucoadhesive patches, which enhance paracellular transport of insulin through iontophoresically induced transient impairment of the tight junction integrity, are also described, and their efficacy has been verified exclusively on animal models, while evidence of the clinical effectiveness of this concept is not yet available [25,98]. Problems that occur due to the use of ingestible devices are mainly connected to limited reservoir capacity, difficult translation of preclinical findings into clinical application, risk of gastrointestinal obstruction and perforation, high production cost, and regulatory issues, as well as the acceptance of these devices by patients [23,64,98]. However, unlike conventional pharmaceutical formulations, ingestible medical devices actively interact with the gastrointestinal environment, representing a paradigm shift from passive drug protection toward active drug delivery. It is expected that advances in robotics and materials used in the development of medical devices will contribute to the creation of systems with greater clinical relevance.

Among ingestible devices, RaniPill^®^ currently demonstrates the greatest clinical potential, having advanced to Phase I clinical trials, while LUMI, SOMA, and intestinal patches remain at the preclinical stage. However, all systems face significant translational challenges, including limited drug-loading capacity, complex and economically demanding manufacturing processes, and safety concerns such as mucosal perforation or gastrointestinal obstruction. Patient acceptability and regulatory barriers further complicate their path toward clinical application. Although RaniPill is presently the most promising candidate, future progress in ingestible devices will depend on simplifying production and reducing cost, establishing suitable evaluation models, and minimising usage-related risks to ensure safety.

#### 4.2.6. Bile-Inspired Strategies

Bile-inspired drug delivery systems represent emerging platforms that have attracted growing interest due to their ability to enhance oral drug delivery. Among novel drug delivery strategies, those utilising bile acids stand out due to the unique properties of these highly valuable constituents, which include their affordability, uncomplicated derivatisation, peculiar physicochemical properties, amphiphilic nature, biocompatibility and ability to act as absorption enhancers [102,129,130,131]. Beyond their physiological role in lipid digestion, bile acids have attracted considerable attention as multifunctional excipients capable of improving oral drug delivery through membrane interaction, modulation of epithelial permeability and exploitation of endogenous transport pathways [129,130,131,132]. For decades, numerous efforts have been undertaken to increase the bioavailability of insulin by combining it with bile acids [129]. Certain bile acid-containing oral drug delivery systems have exhibited promising results in targeting the apical sodium-dependent bile acid transporter (ASBT) located in the ileum, thereby facilitating the cellular uptake of biological therapeutics, including peptides, through ASBT-mediated endocytosis [100,101]. The positively charged bile acid-saturated semaglutide (PBSG) nanocomplex demonstrates activity through a dual mechanism, which is primarily mediated by the presence of bile acids, and it combines both ASBT-mediated drug uptake and ASBT inhibition [100]. This nanocomplex has established itself as a promising candidate for clinical translation due to its improved oral absorption and capability to simultaneously improve glucose regulation, lipid metabolism and liver function [100]. Another promising strategy involves conjugation of a GLP-1 agonist with oligomeric DOCAs (deoxycholic acids), which causes ASBT-mediated endocytosis, improved cellular permeability, and increased oral bioavailability [101]. Research has also shown that deoxycholic acid-modified nanoparticles can increase absorption of encapsulated insulin when administered orally [133]. Although interaction between bile acids and peptide drugs can produce positive outcomes, the potential adverse effects, such as decreased peptide flux across the membrane, must not be overlooked [104]. Such negative outcomes require careful evaluation of both the selected bile acids’ and peptides’ physicochemical properties before their combination [104]. Moreover, bilosomes, biocompatible and biodegradable bile salt-containing vesicular colloidal carriers developed from either liposomes or niosomes, have attracted considerable attention [102,132]. As structural elements, these deformable nano-sized vesicular carriers contain lipids, nonionic surfactants and amphiphilic bile salt molecules [132]. Through the addition of bile salts, such as sodium glycocholate (SGC), sodium deoxycholate (SDC) and sodium taurocholate (STC), into the lipid bilayer, it is possible to prevent further destabilisation of conventional lipid carriers in the presence of intestinal bile salts, gastric acid and pancreatic lipase [134]. Research suggests that bilosomes can increase oral bioavailability by protecting entrapped peptide drugs from the hostile gastrointestinal environment, improving their in vivo resistance, enhancing their permeability across the biological membranes and promoting their transport via the lymphatic system [135,136]. The stability of these systems in a bile salt-rich medium is linked to repulsive interactions observed between bile salts attached to the carrier system and the ones present in the intestine [136]. The benefits provided by the application of these vesicular carriers include greater drug encapsulation efficacy, low toxicity, enhanced selectivity via surface derivatisation, a broad spectrum of therapeutic applications, improved bioavailability through enhanced permeation, stability, non-invasive nature, as well as ability to modulate drug release [102,103]. Despite these advantages, there are also certain drawbacks of these carriers, which are associated with inconsistencies between in vitro and in vivo performance, shortcomings of ex vivo research, challenging encapsulation of anionic compounds due to repulsive interactions, potential cytotoxicity and irritation due to inadequate selection of bile salts [103].

## 5. Regulatory Challenges in Oral Peptide Delivery

Although regulatory frameworks for biological products and conventional oral formulations are well established, specific guidelines from the FDA, EMA, and ICH regarding oral peptide delivery remain generalised and insufficiently comprehensive. Current regulations are applied through broad principles, while the unique challenges associated with oral peptide delivery strategies are often inadequately addressed and remain unconsolidated. Existing guidelines provide a useful foundation, yet their expansion is necessary to offer detailed insight into the actions required prior to registering innovative oral peptide formulations. The definition of peptides varies across regulatory authorities, directly influencing the application of requirements, as no universally accepted definition exists. Existing regulatory frameworks fail to adequately respond to the unique challenges peptides present in drug approval. Although ICH guidelines have contributed to harmonisation, their application to peptide-based therapeutics remains inconsistent and lacks coherence [137]. Structural modifications of peptides can significantly alter receptor-binding affinity, pharmacokinetics, and immunogenic potential and lead to creation of new molecular entities [138]. This underscores the need for rigorous evaluation of their safety and efficacy. Moreover, such modifications introduce greater molecular heterogeneity and elevate manufacturing expenses, thereby posing additional challenges for regulatory approval [138]. EMA provides a regulatory foundation for peptide conjugation and structural derivatisation, highlighting that classification is determined on a case-by-case basis and encouraging early dialogue with regulatory authorities. From this perspective, strict control of starting materials, adherence to GMP during synthesis, and careful evaluation of conjugation-related impurities are required. Such measures ensure that both conjugated and unconjugated species are adequately monitored, safeguarding product quality and consistency. Covalent modifications, in particular, generate new chemical entities requiring comprehensive safety evaluation. Lipidation, as a form of structural modification that can be performed via both covalent and non-covalent methods, exemplifies how scaling up is more demanding when covalent modifications are involved due to the higher heterogeneity of conjugated products, which in turn leads to greater batch-to-batch variability [139]. The process of producing structurally modified peptides must follow universal ICH guidelines for detecting impurities that may be produced during the process. FDA guidelines focus on identifying impurities generated during modification, and when new chemical entities arise, additional safety data are required. Therefore, modified peptides require adherence to stricter CMC and safety standards [137]. General regulatory statements concerning nanomaterials can be applied to nanoparticle systems intended for oral peptide delivery. Given their unique physicochemical properties and interactions with biological systems, regulatory agencies have established frameworks for risk assessment, requiring detailed characterisation of structure, function, ADME properties, material quality, stability, and toxicological profiles. The development of nanoparticles in nanomedicine is hindered by their complex design, unreliable laboratory models, and limited ability to predict biological effects [140]. Batch-to-batch inconsistency and quality variation may arise during the preparation process, as nanoparticles are known to be highly susceptible to external conditions [141]. Regulation of nanomedicine faces several obstacles, including the absence of a standardised definition or classification of nanomaterials, the lack of consistent regulatory frameworks, and the need for analytical methods tailored to each specific material [140]. This methodological heterogeneity further complicates preclinical assessment, reducing standardisation and comparability of outcomes. Additional difficulties arise from pharmacokinetic profiles that differ from conventional substances, as well as stability problems encountered during large-scale manufacturing [140]. Preclinical limitations carry over into clinical translation, where progress is further restricted by limited long-term studies on bioaccumulation, biotransformation, and elimination, as well as by the lack of reliable models that replicate human physiology. A harmonised regulatory framework and consistent definitions are essential to ensure proper categorisation of these nano-sized systems and to secure alignment with regulatory requirements. Further efforts are required to establish reliable models for the assessment of nanoparticle performance. Enzyme inhibitors, mucus-interacting systems, and absorption enhancers may be considered within the framework of regulatory guidelines pertaining to excipients. It is essential that they comply with the general requirements applicable to all excipients, including quality assessment, compatibility, stability, and safety, while also being considered suitable for use. Moreover, adherence to regulatory principles depends on whether these are established excipients or novel entities, since novel entities necessitate a detailed and comprehensive evaluation of safety as well as a rigorous justification for their application. Given their ability to modulate biological functions through mechanisms such as enzyme inhibition, mucus interaction, and alternation of the gastrointestinal epithelium, regulatory agencies should develop specific criteria for such excipients, addressing in detail the evaluation of their safety and justification of their application. Ingestible medical devices for peptide delivery are classified as combination products, integrating device and biologic components. Their evaluation requires thorough assessment of biocompatibility, safety, and device–drug interactions. These active devices must comply with ICH Q8, Q9, and Q10 guidelines, while in the EU they fall under the Medical Device Regulation (MDR). [138] Major challenges encompass the need for reproducible delivery, assurance of safety, and cost-effectiveness of therapy. Essential quality attributes that must be satisfied include ensuring that the device remains intact until reaching the delivery site, reliability of actuation triggers, reproducibility of force generation, precision of delivery, uniformity of drug content, delivered-dose fraction, and completeness of needle actuation [138]. Complex assemblies involving microscopic needles, springs, and electronics require exceptionally precise manufacturing, thereby significantly increasing production costs [138]. Moreover, these systems are not universally applicable: they are unsuitable for drugs requiring high daily doses, medicines with a narrow therapeutic window where variability in delivery could carry a risk for patients, and in conditions where gastrointestinal mucosal integrity is compromised [138]. Rigorous testing is indispensable, yet traditional laboratory models are inadequate for evaluating advanced oral delivery systems. While organ-in-a-chip platforms appear promising, they remain costly and technically demanding [142]. Future progress requires accessible and reliable models that accurately reflect human gastrointestinal physiology.

Regulatory authorities frequently provide only broad frameworks, while explicit and well-defined criteria for the approval of innovative oral peptide drug formulations remain insufficient, which can substantially hinder the pace of development. Overly generalised standards often result in misinterpretations, wasted time, and additional obstacles during the registration process. This lack of specificity slows development, fosters misinterpretation, and complicates registration. Establishing unified, standardised rules and optimising preclinical models are essential to ensure economic feasibility, physiological relevance, and valid results. Coordinated international efforts are required to create harmonised, science-based standards that foster innovation and global progress.

It is also important to emphasise that some of the most successful oral peptide formulations, such oral semaglutide (Rybelsus^®^) and oral octreotide (Mycapssa^®^), achieve bioavailability in the ranges of 0.4–1% and 0.6–0.8%, respectively [58]. Although these values remain low in absolute terms, both formulations have proven clinically effective due to the high potency of the active molecules, where even minimal systemic exposure is sufficient to induce the desired therapeutic response. These examples illustrate that formulation strategies can enhance bioavailability compared to unmodified peptides, yet it remains inherently limited. Consequently, it is essential to establish clear criteria for peptide selection, as not all molecules are suitable for oral delivery [64]. A systematic framework that identifies exclusion parameters would enable sponsors and regulators to triage candidates early in development, thereby conserving resources and focusing efforts on peptides with realistic potential for oral administration [64].

Oral peptide delivery still remains a big challenge since there is no universal formulation approach, but rather each case should be considered individually by taking specific limitations into account. In the absence of a universally superior platform, formulation design remains a meticulous balancing act, as it requires a careful evaluation of the advantages and limitations of each technology in conjunction with the specific peptide payload. To compensate for existing limitations of individual strategies, contemporary research devotes increasing attention to studying combinations of different technological approaches which complement each other. POD™ (Protein Oral Delivery) technology serves as a clear example of a system that integrates several strategies, including penetration enhancers, protease inhibitors and a protective capsule. The synergy of different complementary mechanisms offers a more viable pathway for overcoming existing gastrointestinal obstacles by simultaneously addressing biological barriers at multiple levels. However, many technologies still remain in the preclinical phase, most frequently as a result of the complicated translation of results, potential toxicity, invasiveness, regulatory challenges and high manufacturing costs. Strategies like cationisation and the use of mucolytic agents failed to materialise due to safety issues, as well as certain inorganic compounds whose tendency toward accumulation hindered practical application. The greatest commercial success was achieved through the implementation of milder absorption enhancers, such as SNAC (Rybelsus^®^, Wegovy^®^ pill) and 5-CNAC; different peptide structural modifications (DDAVP^®^, Melt Minirin^®^); self-emulsifying drug delivery systems (Neoral^®^); and certain enzyme inhibitors that enabled progression into late-stage trials and, in some cases, reached the market. However, structural modifications, nanocarriers, absorption enhancers, enzyme inhibitors and ingestible devices also face regulatory challenges, as they create new chemical entities, include complex excipients or combine a drug with a medical device and, consequently, require rigorous assessment of safety and effectiveness. In the case of nanoparticles and ingestible devices, scaling up production to an industrial level is complex and it requires major investments. Although they are financially demanding and require sophisticated manufacturing technology, ingestible medical devices provide a practical means of overcoming key physical and biochemical barriers of the gastrointestinal tract. Ingestible devices based on microneedles that dissolve in contact with the intestinal mucosa minimise invasiveness and point toward a useful direction for improving oral peptide delivery. However, to advance their development, rigorous testing is indispensable. Conventional laboratory models are not suitable for evaluating such complex systems, and while innovative approaches that mimic intestinal function exist, they remain too expensive and technically demanding for routine use.

## 6. Conclusions

Peptide drugs hold a significant place in modern pharmacotherapy, but their broader oral application still remains a big challenge due to low gastrointestinal stability, susceptibility to enzymatic degradation and limited permeability through the intestinal membrane. Consequently, the development of effective oral formulations of peptides represents one of the most prominent research fields in pharmaceutical technology. This review outlines key strategies used for overcoming these obstacles, including structural peptide modifications, enzyme inhibitors, and absorption enhancers, as well as the development of novel drug carrier systems based on lipids, polymers, inorganic nanoparticles, bile acids and salts. Innovative ingestible devices and microneedle systems that allow targeted and effective delivery of peptide drugs through the gastrointestinal tract are of particular interest. A combination of different approaches in one formulation demonstrates remarkable potential for bioavailability enhancement and advancement of the therapeutic effect. Registration of oral formulations of certain peptide drugs, such as semaglutide, offers compelling proof that oral peptide delivery can be successfully implemented in clinical practice. Nevertheless, numerous technologies still remain in the development phase, and their expanded implementation is restricted by safety concerns, manufacturing costs, formulation reproducibility and regulatory requirements. The progress of oral peptide therapeutics is slowed by inconsistent testing, restricted availability of valid preclinical models, and unclear regulatory guidance. These issues collectively create delays, misinterpretations, and difficulties in registration, highlighting the necessity for development of unambiguous and harmonised standards, improved and economically feasible models, and clear systematic criteria for peptide candidate selection. Establishing unified and standardised rules, along with the development of relevant cost-effective preclinical models, is a crucial foundation for economic sustainability, scientific relevance, and reliable outcomes. International collaboration is crucial to establish harmonised, science-driven standards that encourage innovation and enable worldwide advancement. Moreover, forming common rules and practical criteria for peptide selection would help focus efforts on candidates with real potential, making development more efficient. Based on the available evidence, no formulation strategy for the oral delivery of peptides can be considered universally superior or dominant. Each approach possesses distinct advantages and limitations, and its success depends on multiple factors. Consequently, the fact that a particular strategy has enabled the regulatory approval of a peptide-based product or the advancement of certain peptide candidates into late-stage clinical trials should not be interpreted as evidence of its overall superiority. Rather, it reflects its suitability for the specific peptide and therapeutic context. Therefore, no universal solution for oral peptide delivery exists, and each peptide should be evaluated on a case-by-case basis to identify the formulation strategy that best matches its unique characteristics.

### Future Perspectives

Future advances in oral peptide delivery are expected to rely on the rational integration of complementary technologies. The convergence of peptide engineering, advanced pharmaceutical carriers, bioinspired systems and smart ingestible medical devices may ultimately enable the development of clinically translatable oral peptide formulations with improved efficacy, safety and patient acceptability. Furthermore, it is also expected that further development in the field of nanotechnology, biomaterials, medical devices and rational molecule design will lead to the development of a greater number of oral peptide drugs. This would significantly increase patient adherence, simplify the treatment of chronic illness and eventually expand the importance of this promising therapeutic group. In addition, artificial intelligence (AI) and machine learning (ML) approaches may further accelerate the development of oral peptide therapeutics. Recent models have demonstrated the ability to predict peptide stability under gastrointestinal conditions and membrane permeability across different biological barriers, thereby supporting early candidate selection and rational structural optimisation [143,144]. More advanced multimodal models have also shown promising performance in predicting the permeability of cyclic peptides and identifying structural features associated with improved membrane transport [145]. In the future, integration of these computational approaches with formulation development and Quality-by-Design workflows could facilitate excipient selection and optimisation of formulation composition, critical material attributes and process parameters [146]. Nevertheless, peptide-specific formulation datasets remain limited and heterogeneous, and model performance may decline when applied to structurally novel peptides or complex in vivo conditions. AI-based predictions should therefore be used to prioritise experiments and support formulation design, while biorelevant experimental testing and clinical validation remain indispensable.

## Figures and Tables

**Table 1:** Commercially available peptide drugs and peptide-based drugs for oral administration with systemic absorption or local gastrointestinal/oropharyngeal action.

	Peptide Names and Their Amino Acid Sequences/Chemical Compositions	Manufacturer	Indications and Site of Action	Pharmaceutical Form for Oral Administration	Year of Approval
**Orally administered peptides with systemic effect**					
Rybelsus^TM^ Wegovy^®^ pill	semaglutide (H-His-Ala-Glu-Gly-Thr-Phe-Thr-Ser-Asp-Val-Ser-Ser-Tyr-Leu-Glu-Gly-Gln-Ala-Ala-Lys-Glu-Phe-Ile-Ala-Trp-Leu-Val-Arg-Gly-Arg-Gly-OH)	Novo Nordisk (Bagsværd, Denmark)	Type 2 diabetes mellitus (Rybelsus^TM^) (systemic effect); obesity (Wegovy^®^ pill) (systemic effect)	tablets	2020 (Rybelsus^TM^) (EMA, FDA) 2025 (Wegovy^®^ pill) (FDA)
ICOTYDE^TM^	icotrokinra (Ac-cyclo[Pen-Asn-Thr-7MeTrp- Lys(Ac)-Pen]-Phe(2ae)-2Nal- ThpGly- Glu-Asn-3Pal-Sar-NH2)	Johnson & Johnson (New Brunswick, NJ, USA)	Moderate to severe plaque psoriasis (systemic effect)	tablets	2026 (FDA)
Neoral^®^ Sandimmune^®^	cyclosporine (cyclo[Abu-Sar-N(Me)Leu-Val-N (Me)Leu-Ala-D-Ala-N(Me)Leu-N(Me)Leu-N(Me) Val-N(Me)Bmt(E)])	Novartis AG (Basel, Switzerland)	Suppression of immune response; prevention of transplant rejection (systemic effect)	soft capsules	1995 (Neoral^®^) 1990 (Sandimmune^®^)
DDAVP^®^ Tablets Melt Minirin^®^	desmopressin acetate hydrate (deamino-Cys(1)-Tyr-Phe-Gln-Asn-Cys(1)-Pro-D-Arg-Gly-NH2)	Ferring Pharmaceuticals Generic (Saint-Prex, Switzerland)	Neurogenic diabetes insipidus; primary nocturnal enuresis; nocturia associated with multiple sclerosis (systemic effect)	tablets; oral lyophilisate	1995 (DDAVP^®^) 2005 (Melt Minirin^®^)
Ceredist^®^ Ceredist OD^®^	taltirelin hydrate * (thyrotropin- releasing hormone analogue; synthetic oligopeptide composed of three main units- a modified pyrimidine ring, L-histidine, and L-prolinamide, all bonded sequentially)	Mitsubishi Tanabe Pharma Corporation (Osaka, Japan)	Spinocerebellar degeneration; ataxia (systemic effect)	tablets; orodispersible tablets	2000 (Ceredist^®^) 2009 (Ceredist OD^®^)
Reduced L-Glutathione	glutathione (H-gGlu-Cys-Gly-OH)	Theranaturals Inc. (Williston, ND, USA)	Cystic fibrosis; HIV-related cachexia (systemic effect)		
Mycapssa^®^	octreotide (somatostatin analogue) (H-D-Phe-Cys(1)-Phe-D-Trp-Lys- Thr-Cys(1)-Thr- OH)	(Chiasma, Needham, MA, USA)	Growth hormone secretion disorder, acromegaly and neuroendocrine tumours (systemic effect)	delayed-release capsules	2020
**Orally administered peptides with local gastrointestinal/oropharyngeal effect**					
Linzess^®^ (USA) Constella^®^ (Europe)	linaclotide (H-Cys(1)-Cys(2)-Glu-Tyr-Cys(3)- Cys(1)-Asn-Pro-Ala-Cys(2)-Thr-Gly-Cys(3)-Tyr-OH)	Acatavis, Inc. (New Jersey, USA)/Ironwood Pharma, Inc. (Boston, MA, USA)	Local effect in irritable bowel syndrome and chronic idiopathic constipation (local gastrointestinal action)	capsules	2012 (Linzess^®^) (FDA) 2012 (Constella^®^) (EMA)
Vancocin^®^	vancomycin HCl * (complex glycosylated and cross-linked heptapeptide containing non-proteinogenic amino acid residues)	ANI Pharmaceuticals, Inc. (Baudette, MN, USA)	Acts locally on Staphylococcus aureus and Clostridium difficile, leading to selective gastrointestinal decontamination (local gastrointestinal action)	capsules	1986 (FDA)
Koolistin^®^	colistin sulfate * (cyclic polypeptide antibiotic composed of Polymixines E1 and E2)	Biocon Ltd. (Bengaluru, India)	Produces a local effect in intestinal infections caused by susceptible G(-) microorganisms (local gastrointestinal action)		
Several different products	tyrothricin * (antibiotic peptide mixture of linear pentadecapeptides–gramicidines and cyclic decapeptides–tyrocidines, containing both L and D-amino acids)	Various manufacturers	Local treatment of pharyngitis (local oropharyngeal effect)	lozenges	
Trulance^TM^	plecanatide (H-Asn-Asp-Glu-Cys(1)-Glu-Leu-Cys(2)-Val-Asn-Val-Ala-Cys(1)-Thr-Gly-Cys(2)-Leu-OH)	Synergy Pharmaceuticals Inc. (New York, NY, USA)	Local therapy of chronic idiopathic constipation (local gastrointestinal action)	tablets	2017 (FDA)

Source: [2,23,29,31,32,33,34,35,36], supplemented with additional data from [37,38,39,40,41,42,43,44,45]. * Conventional single amino acid sequence not applicable.

**Table 2:** Strategies for oral peptide delivery along with their advantages and limitations.

Strategy				Advantages	Limitations	Development Stage (Exemplified)
**Structural modifications of peptides**	cyclisation			Improves stability, reduces susceptibility to degradation, and increases permeability [21,23,25,34,35,59,60]	It can interfere with biological conformation and reduce biological activity [23].	Cyclic peptides such as cyclosporine A and desmopressin are also registered for oral administration [30].
	PEGylation			Reduces renal clearance, inhibits enzyme access and thereby increases stability in circulation, masks antigenic epitopes, and promotes diffusion through the mucus barrier [6,23,25,53,60,61]	Extensive PEGylation can interfere with peptide binding to receptors and reduce biological activity [6,7].	The PEGylated insulin analogue from the Indian company Biocon (Insulin Tregopil IN-105) has completed Phase II/III clinical trials [30,62].
	lipidation			Lower hydrophilicity, longer plasma half-life, and avoids the first-pass effect [21,23,60,63]	Reduces peptide water solubility, decreases affinity for relevant receptors [7].	Semaglutide was generated by acylation of natural GLP-1 and is registered for oral administration [64].
	natural L-amino acid substitution			It reduces the susceptibility to enzymatic degradation [21]	It can alter conformation and efficacy of peptides [65]. A potential risk of immunogenicity and toxicity arises [66].	In the structure of vasopressin, L-arginine is substituted with D-arginine, resulting in the formation of desmopressin, a peptide whose oral formulations are registered [2].
	N-methylation			It protects from enzymatic degradation and increases resilience to proteolysis [6,65]	The production process is complex, and side reactions may occur [67].	By N-methylation of vasopressin, desmopressin was developed, which has been registered for oral administration [6].
	cationisation			It enhances permeation through intestinal mucosa [7,65,66,68]	Increased immunogenicity, accelerated clearance, loss of drug activity, nonspecific cellular uptake, and potential toxicity [7].	Due to potential toxicity, it has no clinical application [7].
	prodrug design			Increased stability, solubility, lipophilicity and intestinal permeability [7,63]	Potential toxicity of the bound moiety [6], limitations regarding stability, and structural complexity may cause problems during design process [23].	TPLENK is an experimental, lipophilic prodrug of encephalin tested on animal models [6] (preclinical stage).
**Enzyme inhibitors**	aprotinin, FK-448, soybean trypsin inhibitor, leupeptin, ovomucoids [21,26,32,34]			Inactivation of proteolytic enzymes and protection of peptides from degradation [23,32]	Interferes with the digestion of dietary proteins; can cause malabsorption, hypertrophy and hyperplasia of the pancreas [2,33,34,69].	ORMD-0801 is an oral insulin formulation that is in Phase III clinical trials. It is based on POD™ technology that combines several formulation strategies, including enzyme inhibitors such as soybean trypsin inhibitor and aprotinin [7,28,62,70]. Despite demonstrating feasibility, Oramed’s ORMD-0801 achieved mixed results in Phase III studies, illustrating the translational challenges associated with its clinical development [71].
**Absorption enhancers**	surfactants, fatty acids, bile salt derivatives, chelating agents, chitosan, cholesterol, glycerides, salicylates, nitrogenous-containing heterocyclic compounds, cell-penetrating peptides (CPPs), aromatic alcohols [23,49,72]			Enhancing transcellular or paracellular delivery of peptide drugs [25,26]	Prolonged and uncontrolled use can lead to membrane damage, inflammation of the gastrointestinal tract and endotoxemia [25,26,34,69,73].	In preclinical trials, numerous absorption enhancers were tested, and only some demonstrated an appropriate safety and efficacy profile and entered clinical trials [25]. The registered oral formulation of semaglutide-Rybelsus^®^ contains the SNAC penetration enhancer [4,22,34]. Mycapssa^®^, a registered oral formulation of octreotide, is based on TPE^®^ (engl. Transient Permeation Enhancer) technology, whose main absorption promoter is Na-caprylate [4,34,64]. 5-CNAC was in Phase III clinical trials as a permeability enhancer for the oral formulation of salmon calcitonin, but the results of the evaluation of safety and efficacy did not meet expectations [33,74]. ORMD-0801 is an oral insulin formulation that is in Phase III clinical trials, and is based on POD™ technology, which is a combination of several formulation strategies, including absorption enhancers such as EDTA and bile salts [7,28,62,70]. Despite demonstrating feasibility, Oramed’s ORMD-0801 achieved mixed results in Phase III studies, illustrating the translational challenges associated with its clinical development [71].
**Mucus-interacting systems:**	mucolytic agents (dithiothreitol, N-acetylcystein, bromelain, papain) [23]			Cleavage of the intermolecular disulfide bonds of the mucus barrier increases oral bioavailability [23,36,63]	Uncontrolled removal of the protective mucus layer exposes the mucosa to harsh gastrointestinal conditions [23,36,63].	They have not been validated in clinical trials despite numerous studies [7].
	mucus-penetrating systems (nanoparticles coated with biocompatible polymers such as PEG, chitosan + alginate, and poloxamer) [75,76]			By modifying the surface of drug-loaded nanoparticles through addition of nonionic polymers or by coating them with a dense, homogeneous layer with equilibrated opposite charges, electrostatic interactions with mucus are reduced, allowing enhanced penetration across this barrier [75]	These systems are confronted with certain challenges regarding manufacturing cost, drug-loading capacity, scalability, clearance and potential toxicity [77].	Mucus-penetrating insulin-loaded poly(n-butylcyanoacrylate) nanoparticles coated first with chitosan and then with alginate (Ins/PBCA/CS/Alg NPs) were examined in vivo in diabetic rats for oral insulin delivery [76] (preclinical stage). Following multiple oral administration, mucus-penetrating cyclosporine A-loaded PEG-PLGA (poly(ethylene glycol)-block-poly(lactic-co-glycolic acid) nanoparticles demonstrated anti-inflammatory effects in the model rats [78] (preclinical stage).
	mucoadhesive systems (nanoparticles coated with mucoadhesive polymers such as chitosan, polymeric drug delivery systems (polymeric nanoparticles and hydrogel formulations) and intestinal patches with mucoadhesive properties)			Mucoadhesive properties increase residence time [75,79]	Insufficient adhesion to the mucus may result in displacement due to gastrointestinal motility and mucus turnover [75,79].	Mucoadhesive insulin-loaded poly(n-butylcyanoacrylate) nanoparticles coated with chitosan (Ins/PBCA/CS NPs) were examined in vivo in diabetic rats for oral insulin delivery [76] (preclinical stage). See the sections of this table dedicated to polymeric nanoparticles, hydrogel formulations, and intestinal patches for further information.
**Drug delivery systems**	lipid drug delivery systems	lipid nanoparticles (liposomes, SLNs, NLCs) [6]		The lipid composition of these nanoparticles promotes absorption into the lymphatic system; they are biocompatible, biodegradable, and they protect encapsulated peptides [6,32,36,80,81,82,83]	Susceptibility to oxidation, difficulties in industrial production, tendency to aggregate, thermodynamic instability, and problematic stability in the presence of salts and enzymes and with changes in pH value [6,7,84,85].	Preclinical studies generally showed improved stability and a moderate increase in absorption; however, the attempt to clinically translate these results was not successful [64] (preclinical stage).
		SEDDSs (SNEDDSs, SMEDDSs) [21,49,70]		Simple and cost-effective production, increases drug permeation through the mucus barrier, and protects peptides from presystemic metabolism [6,21,52,70]	Liquid SEDDSs can exhibit phase separation, precipitation, or leakage throughout the storage period [86].	SEDDS technology was used in the formulation of the oral form of cyclosporine A, registered under trade name Neoral^®^ [70].
	polymeric drug delivery systems	polymeric nanoparticles (PLGA, PLA, PHA, polysaccharides (alginate, chitosan)) [6]		Polysaccharide nanoparticles are biocompatible, biodegradable, and safe; they enable pH-responsive drug release, can interact with mucus, and extend residence time [6,21,87,88]	Polymeric drug delivery systems show a tendency to aggregate, have a small drug-loading capacity and face challenges in scaling up the manufacturing process to an industrial level [6].	Preclinical studies generally showed improved stability and a moderate increase in absorption; however, the attempt to clinically translate these results was not successful [64] (preclinical stage).
		hydrogel [25]		Controlled release [7]; pH-responsive alginate and chitosan-based hydrogels are non-immunogenic, and they do not require drastic conditions for peptide incorporation [89,90,91]	Rapid degradation upon contact with large amounts of gastrointestinal fluid, and physical and chemical instability [7].	Hydrogels have not reached clinical trials [7] (preclinical stage).
	inorganic particles (silica, selenium, gold, titanium dioxide, zirconium phosphate, calcium phosphate, carbon) [21,32,92,93]			They are stable in acidic and enzyme-rich medium [21]. Some of these carriers are biocompatible with tunable physicochemical properties, with the ability for precise control of shape and size and the potential for surface modifications [6,84,93,94]	Slow and incomplete in vivo degradation accompanied by the risk of tissue accumulation [32,63].	Oshadi Drug Administration has developed silica-based nanoparticles for insulin delivery, which have successfully passed Phase II clinical trials [32,62]. The endocytosis of various inorganic nanoparticles has been investigated in vitro, ex vivo and in vivo [95].
	smart ingestible medical devices	microneedle devices	LUMI [96,97]	Biodegradable ingestible devices provide protection and enable the delivery of intact peptides [7,70]	The approach can be invasive and may increase the risk of mucosal perforation; it has a small drug encapsulation capacity, it is complex and expensive to manufacture and the effectiveness depends on interpersonal variations in peristalsis [7,64,70]. Problems associated with the use of ingestible devices include limited reservoir capacity, problematic translation of preclinical results, risk of gastrointestinal obstruction and perforation, regulatory issues and problematic acceptability among patients [23].	No confirmation of clinical efficacy; still in the preclinical stage [7]. RaniPill^®^ is being investigated for the oral delivery of octreotide and it has completed Phase I clinical trials [62,70].
			SOMA [96,98]			
			RaniPill^®^ [70,84]			
		intestinal patches and microreservoirs [23]		They protect the incorporated drug from degradation and contribute to positioning the drug close to the absorptive surface [7,25,99]	Limited capacity for drug incorporation and reduced stability throughout the storage period [7]. Intestinal mucoadhesive patches that are too thick can potentially detach and interfere with fluid transit [32].	The effectiveness has so far been confirmed exclusively in animal models, while evidence of clinical efficacy is not available yet [23,25] (preclinical stage).
**Bile-derived drug delivery systems**		bile acid-saturated nanocomplex [100]; conjugation with oligomeric DOCAs [101]; bilosomes [102]		Improved cellular permeability, along with increased oral bioavailability [101]; greater drug encapsulation efficacy, low toxicity, enhanced selectivity via surface derivatisation, broad spectrum of therapeutic use, improved bioavailability through enhanced permeation, stability and non-invasive nature, as well as ability to modulate drug release [102,103]	The interaction between the peptide and bile acid can decrease peptide flux across the membrane [104]. Issues: inconsistency between in vitro and in vivo performance, shortcomings of ex vivo research, challenging encapsulation of anionic compounds due to repulsive interactions, potential cytotoxicity and irritation due to inadequate selection of bile salts [103].	Still in preclinical stage [101,105].

## Data Availability

No new data were created or analysed in this study. Data sharing does not apply to this article.
